# Exploring the views of GPs, people with dementia and their carers on assistive technology: a qualitative study

**DOI:** 10.1136/bmjopen-2016-011132

**Published:** 2016-05-13

**Authors:** Lisa Newton, Claire Dickinson, Grant Gibson, Katie Brittain, Louise Robinson

**Affiliations:** 1 Institute of Health & Society, Newcastle University, Newcastle upon Tyne, UK; 2 School of Applied Social Sciences, Colin Bell Building, University of Stirling, Stirling, Scotland

**Keywords:** QUALITATIVE RESEARCH, PRIMARY CARE, GERIATRIC MEDICINE

## Abstract

**Objectives:**

To explore the views and experiences of people with dementia, their family carers and general practitioners (GPs) on their knowledge and experience of accessing information about, and use of, assistive technology (AT) in dementia care.

**Design:**

Qualitative methods with semistructured interviews and thematic analysis.

**Participants:**

56 participants comprising 17 GPs, 13 people with dementia and 26 family carers.

**Setting:**

Community care settings in the North East of England.

**Results:**

4 main themes emerged: awareness and experience of AT; accessing information on AT; roles and responsibilities in the current care system and the future commissioning of AT services. All participants had practical experience of witnessing AT being used in practice. For people with dementia and their families, knowledge was usually gained from personal experience rather than from health and social care professionals. For GPs, knowledge was largely gained through experiential, patient-led learning. All groups acknowledged the important role of the voluntary sector but agreed a need for clear information pathways for AT; such pathways were perceived to be essential to both service providers and service commissioners.

**Conclusions:**

People with dementia and their family carers appear to be mainly responsible for driving a gradual increase in both awareness and the use of AT in dementia care. GPs should be equipped with the relevant knowledge to ensure families living with dementia receive appropriate information and support to enable them to live independently for as long as possible. There is an urgent need to simplify current complex community care pathways; as demonstrated in other chronic health conditions, a single point of access and a named lead professional may improve future care.

**Strengths and limitations of this study**

- Assistive technology (AT) has the potential to enhance patient independence and safety, especially for those living with dementia. In the UK, there has been a longstanding policy push for health and social care to drive implementation. Qualitative research allows an in-depth exploration of the facilitators and barriers which influence uptake of such a complex intervention.
- This study explored the views and experiences of both ‘users’ (people with dementia and their family carers) and service providers (general practitioners (GPs)) about how, and if, AT is used in dementia care.
- Data was collected during a period of considerable re-organisation in the health service in England; the introduction of GP-led clinical commissioning. Participants included both GPs with service commissioning roles as well as service provider responsibilities.
- However, there are limitations. The study was conducted in one geographical region of England. The results may not be generalisable to other areas, as access to AT may vary between different regions. Additionally only one health professional group were sampled; the perspectives of the wider dementia care team should be sought.

## Background

Globally the number of people with dementia, and the associated costs of their care, are predicted to increase yet there is still much to be done to improve the quality of dementia care.^1^ Considerable geographical inequalities in the provision of post-diagnostic services exist despite a policy push in the UK towards more timely diagnosis and earlier intervention.^2^ Assistive technology (AT) is one aspect of post-diagnostic care, which may enable people with dementia to remain independent for longer, thus potentially leading to cost savings by delaying entry into care homes.^2^ ^3^

AT encompasses a wide spectrum of technological solutions to problems people encounter in their everyday lives. The term generally includes telecare and telehealth but can also incorporate simple aids/devices, such as a walking stick or hearing aid. A number of devices have been specifically developed for people with dementia to enhance their safety, aid communication and facilitate leisure activities. Evidence suggests that people with dementia are positive about using AT.^4^ However evidence for its clinical and cost-effectiveness is limited.^5^ ^6^ While a large randomised controlled trial is underway in the UK to address this very question,^7^ small studies to date have found AT improved independence^8^ and problem behaviour in people with dementia^9^ and for their carers, improved quality of life^8^ and reduced stress.^9^ However, while large randomised trials are essential to evaluate effectiveness, parallel qualitative studies facilitate an understanding about how such complex interventions can be successfully implemented in practice.^10^

People with dementia and their carers require reliable, accurate, and up to date information in order to access AT. In England, health and social care websites and voluntary organisations, such as the Alzheimer's Society, highlight that general practitioners (GPs) should be able to refer on for further information and assessment in relation to AT.^11^ Recent research explored GPs' views on the role of telehealth in the management of long-term conditions and found a lack of enthusiasm among GPs around the use of telehealth, with concerns as to how it would impact on their workload.^12^ ^13^ In England, some GPs are now involved in commissioning healthcare services as well as providing care. This study aimed to explore the views and experiences of GPs, people with dementia and their family carers on their knowledge and use of AT in practice.

## Methods

The initial approach regarding information about the study and an invitation to participate was by email for GPs and by letter to people with dementia and their family carers. One of three experienced qualitative researchers (CD, GG and KB) and one Master's student (LN) conducted semistructured interviews at a site of the participants' choosing. We purposively sampled to ensure a range of participants. We sought people with dementia and carers who had experience of AT through statutory services and non-statutory settings and those of different ages; we also sought GPs, GP trainees and GPs with a commissioning role. Participation was voluntary. No participants dropped out of the study. All but one GP was interviewed at their place of work; people with dementia and carers were interviewed at a place of convenience to them, with the majority being interviewed at home. Interviews lasted approximately 1 h and were conducted between September 2013 and November 2014. Researchers ensured people with dementia had capacity to take part and gained written informed consent from all participants before data collection started.

We developed interview schedules for each participant group from a literature review^14^ and subsequently refined these following initial pilot interviews. We hypothesised that many participants would have a low level of awareness around existing devices, therefore a selection of photographic images of AT used by and with people with dementia were shown to participants to facilitate discussion (box 1). Researchers asked participants about their experience of AT and encouraged them to give practical examples. Topic areas explored during interviews are outlined in table 1.

Box 1 **Types of assistive technology which were shown to participants in photographic images**

- Community alarms and telecare.
- Global positioning system (GPS) location monitoring devices.
- Signage.
- Reminiscence tools.
- Clocks to aid orientation.
- Simplified telephones with pictures.
- Dementia friendly furniture.

**Table 1:** Topic areas explored within individual participant interviews

GP Interviews	Knowledge and experience of technology to assist people with dementia. Experiences of accessing information and giving information to people with dementia. Knowledge and experience of referring people with dementia for AT. Their views on commissioning AT for people with dementia.
People with dementia and carers	General feelings about the use of technology to assist people with dementia. Views about speciality designed AT used in dementia care (via demonstration photographs). Current use or non-use of AT as everyday technology and specially developed AT. Experiences or accessing both information about AT and specific devices. Views regarding the use of or non-use of AT as dementia progress.

AT, assistive technology; GP, general practitioner.

All participants were recruited from the North East of England. GPs were recruited from a range of local sources: the primary care research network, the GP vocational training scheme and Clinical Commissioning Groups (CCGs). They worked in areas covering four CCGs. People with dementia and carers were recruited from: local dementia cafes, a day centre, a local authority telecare service, a GP surgery and a public participation in research forum. We digitally recorded all interviews and they were transcribed verbatim. Transcripts were analysed using thematic analysis.^15^ LN (GP data) and GG (people with dementia and carer data) initially read transcripts and coded line by line to identify initial themes. The wider team (LR, CD, GG and LN) then considered the initial themes looking for areas of overlap and discrepancy between the different groups of participants. NVivo software was used to manage data.

## Results

We interviewed a total of 56 participants; 17 GPs (including 6 GP trainees and 5 GPs with a commissioning role), 13 people with dementia and 26 carers (18 current and 8 former carers). People with dementia were aged between 49 and 91 years (mean age 72 years) and carers were aged between 49 and 82 years (mean age 61 years). GPs were aged between 34 and 58 years (mean age 42 years) and GP trainees were aged between 27 and 34 years (mean age 30 years).

Four major inter-related themes emerged from the data:

- Awareness and experience of AT;
- Accessing information on AT;
- Roles and responsibilities in the current care system;
- Future commissioning of AT services.

### Awareness and experience of AT: ‘technology what the h--- does that mean?’

Carers and GPs generally found the term AT unhelpful and open to interpretation. GPs identified the term ‘technology’ as being potentially difficult for older people, who they assumed may lack knowledge or have preconceived ideas about technology. Some people with dementia also agreed they found the concept of technology challenging.

Well, I think the whole thing was introduced to me in a very nebulous way. Technology, what the hell does that mean? (Carer 14)

I'm actually not 100% clear. I guess it could be anything which makes life easier for people with dementia, so kind of simple aids around the house. (GP 12)

I think your ability to absorb it has a cut-off point and it's no good fighting against it. And I've just hit the buffers as it were with the technology. (Person with dementia 12)

All participants had personal, and sometimes practical, experience of a range of devices. Examples included those used to: maintain safety (pendant alarms, fall alarms, door exit sensors); help orientation (signage, clocks and notice boards), alert people with dementia or carers (pill dispensers) and facilitate communication (easy to use telephones). Most GPs gained their knowledge experientially, either during their training or through visiting patients at home or in care homes.

…medication prompts, where you have the days of the week on a medibox…so it's really things like that, to help them get through their day-to-day life, whether with medication, or keeping them safe with pendant alarms and things for falls. (GP 8)

People with dementia and their families also had experience of seeing AT in use in care settings such as residential homes and hospitals, when visiting older family members or friends. These devices tended to be pendant alarms and examples of telecare. Family carers played a key role in ensuring implementation of AT within the person with dementia's daily routine, either purchasing devices or adapting existing equipment to create individually tailored AT to assist their relatives.

Yes, well, because the sheltered housing schemes that I’ve managed have always had these [referring to the range of AT devices shown in photographic images]… it's not just about mental health problems. (Person with dementia 22)

“I see them on the TV and I would buy them [household devices such as movement sensors]. Nobody's said anything… she was leaving the gas on so I decided to cut the gas out altogether except for the heating. (Carer 7)

While participants appeared to have experience of using a wide range of AT in real-world settings, the means by which they accessed information on how to acquire such devices proved less than straightforward.

### Accessing appropriate and reliable information on AT: ‘I'm sure there's nothing on AT’

None of the GP participants had ever sought information on AT for a patient or had a patient with dementia or carer approach them for such advice. In terms of where they would seek information about AT, GPs listed a range of potential sources including other professionals, local and national voluntary organisations and online websites; however the primary care websites they generally used as an information resource did not include AT information or the intranet used by GPs with a commissioning role.

I'm not quite sure what I got on web Mentor [a software package used by GPs]… the one I use virtually all the time. (GP 10)

We tend to have our own network, a portal of information system as well. And I'm the one that deals with the dementia. And I'm sure there's nothing on AT. (GP 9; Commissioning role)

In terms of consulting other professionals, most GPs stated they would wish to consult an ‘expert’, who had up to date knowledge of AT but most GPs were unclear who this expert would be. Suggestions included occupational therapists (OTs), specialists in older people's services, nurses based in memory assessment services and those working in community settings. GPs felt that electronic information sources should be trustworthy and impartial; many listed voluntary organisations such as the Alzheimer's Society and Age UK, in addition to National Health Service (NHS) websites, while two mentioned specific AT websites. Most were reluctant to use other online resources as they were unsure of their reliability in recommending safe and cost-effective devices.

But if it was specifically for a patient then I'd go to the, sort of, probably the OTs and the DNs [district nurses] and things, or care of the elderly physicians. …I would assume that they would have better knowledge about than I, and also access to providing them. (GP 15; Trainee)

And I want them to go to the right place. So if I do anything online, it would be Alzheimer's Society …because I mean it's trusted…I know they are more specific for dementia. (GP 9; Commissioning role)

People living with dementia shared professional uncertainties about how to locate information on AT via existing health and social care pathways and also emphasised the importance of voluntary sector organisations such as the Alzheimer's Society or Age UK. Such resources were trusted sources of information based on their capacity to provide ‘unbiased’ advice not motivated by the desire to make a profit from the sale of products or services. Local initiatives for people with dementia, such as peer support groups and dementia cafes, were an opportunity for people to actually try out AT products and devices. However, while the voluntary sector was seen to play a crucial role in providing trusted information, people with dementia and their carers still felt their services could be improved with a request for them to provide more comprehensive and detailed information about AT products and services.

INT: Has your GP ever told you anything about …

PWD: Well not so much, no not really. I think we only get it from, from the dementia cafe. (Person with dementia 26)

I think there is not enough information given out… You know, if there were leaflets saying facilities for Alzheimer's patients, websites. I think people would go on them. (Carer 4)

Participants reported an absence of clear information pathways. This was mirrored by a similar issue around lack of clarity around the specific roles and responsibilities of the wide range of professionals often involved in providing care to families living with dementia.

### Roles and responsibilities in the current care system

#### The role of the GP: ‘it's not on my radar’

Many GPs felt that AT did not come into their consciousness when they reviewed their patients with dementia. They felt it was not their role to assess patients for AT as they would not have up to date knowledge on the products, and any associated costs, in a rapidly changing field. Most GPs did however feel they had a clear responsibility to signpost patients for further information or to refer them to a specialist service for AT assessment.

I very rarely think, “What could we put in place that would make this person's life easier?” …it's not on my radar when I look and review dementia patients from a health perspective. (GP 7; Commissioning role)

Pointing in the right direction and/or their carer in the right direction, because like any specialist services, it's just going to change and having an idea and the resources to point them in the right direction is probably where I would see our role. (GP 8; Trainee)

GPs acknowledged AT was not a core part of their dementia care. However, they did comment on their extensive role in caring for people throughout the illness trajectory from facilitating the diagnosis to managing the chronic illness and any associated acute illness and crisis events. Most acknowledged they lacked time to talk about AT with people with dementia

I look at their chronic disease, their mental health, how they're functioning, carer support; I look at advance care planning. But actually how we use AT is not and maybe it should be” (GP 7; Commissioning role)

Time is always a constraint…trying to deal with them in a crisis …a lot of this needs to be done in a more proactive way. (GP 16; Commissioning role)

People with dementia and carers also perceived that their GPs' time was precious and should be spent managing physical health concerns rather than issues relating to everyday living. Generally they felt that GPs would not be able to provide up to date information on AT. However, people with dementia and their carers considered some GPs to be much more ‘dementia friendly’ and thus perceived as more approachable and interested.

I've had absolutely no luck with his GP. He's perfectly pleasant but he's not terribly helpful, he's not into Alzheimer's. My GP is very nice and she's much more interested in [husband] than he is. (Carer 3)

#### A diffusion of responsibility: “we all seem to do a little bit of dementia”

Most participants were unclear who had responsibility for giving information and/or providing AT to patients and whether it ultimately lay with primary, secondary or social care. None of the GPs had referred people with dementia for a general AT assessment and only two had referred patients directly for AT (pendant alarms); one GP had signposted patients to a charitable organisation for telecare access. Several GPs reflected why dementia was perceived to be different to other chronic diseases in that there appeared to be a shared responsibility for many areas with an absence of a lead professional or key worker to co-ordinate care between different professional groups.

We all seem to do a little bit of dementia each, but we don't have perfectly dedicated people and if we do they're very secondary care positioned. (GP 16; Commissioning role)

To be honest it's probably quite shameful but once they've been referred to the memory clinic, I've always just presumed they go out and do the assessments and decide what home aids they need. (GP 13)

People with dementia and family carers also identified the lack of clear lines of responsibility among care providers but this concern extended beyond merely AT and to post-diagnostic care generally with many feeling unsupported for considerable periods of time after getting their diagnosis.

Just shove a few more pills down you and leave you for another three months and that's about where we're up to at the moment. We haven't seen anybody for nearly two and a half months…at the moment we're just about coping. (Person with dementia 26)

However, several GPs suggested these issues of responsibility were not insurmountable and provided practical solutions including a dementia nurse specialist to act as a key worker and introduction of clear referral pathways.

When it's COPD or heart failure and things like that and you know, “Oh maybe we'll think about something telehealth, is there something?” And a speciality nurse is linked with that. That would probably prompt you …But when you think of dementia it's probably a lot wider…we don't really have dementia nurses. (GP 16; Commissioning role)

I think having a clear pathway. Actually having a set of things that are readily made, that you know cover different areas where they improve health, and knowing exactly where to go to. Single points of contact are really useful (GP 16; Commissioning role)

Participants reported a lack of clarity within information networks and current care systems. This combined with a perceived diffusion of responsibility for dementia care was also influential in the final theme, the future commissioning of AT services.

### Future commissioning of AT: ‘there are huge gaps within existing services’

Participants highlighted the governance and monitoring of AT services as a key issue, as GPs in England now have responsibility for commissioning clinical services. Wider implementation issues, such as who should respond to alerts, maintain devices and train people to use AT frequently went unaddressed. Doctors with commissioning roles reflected on gaps within relevant community systems, (health, social care, emergency and police services), which could prevent an appropriate response to AT systems.

…but there's huge gaps within existing services… sometimes the use of this [AT] clearly points out the gaps within the sort of services that you need to think about [the] redesign of. (GP 16; Commissioning role)

Most GPs believed AT should be commissioned by social services, a few felt it should be a joint health and social care issue. GPs also identified that AT may not be a priority for dementia care commissioning as there may be more ‘important’ competing priorities such as the current policy driven agenda to increase diagnosis rates.

I think if it's perceived as a health technology, then effectively it is a health but then when it's perceived as this keeps someone in their own home. So that would come as a social care…at the minute we're measured against our peers on how many of our patients have diagnosis…It's like the government sets these targets and we sort of work our way through it. (GP 7; Commissioning role)

One GP commented that the focus of dementia care was still deeply rooted in a ‘medical model’; such an emphasis on ‘cure not care’ led to non-drug interventions such as AT often being neglected.

You need somebody who will actually give them practical tips and advice, who will actually let them cry on their shoulder, And that's where I think we, as society, let patients down, because we all collude with each other into thinking that medicine, medicine, medicine has the answer. And actually, care has the answer, until such time as we find a cure, or a preventative thing for dementia…. And if devices like this help, both—either—or the carer and the patient, then that's what we should be doing… (GP 1)

People with dementia and their carers expressed opinions on future dementia services. They acknowledged the rising role of GPs and the wider role of primary care in the care of an ageing population. A number of participants felt that primary care was an ideal setting to deal with AT and called for closer working between primary care and social services.

I don't know what is out there but your GP has a certain responsibility as well, and especially now, I mean, I know there's a big thing at the moment about the population getting older and older and older. (Carer 16)

Not so much your GP, but perhaps the surgery should have details, social services should be pointing you in the right direction. (Carer 20)

Participants identified a number of perceived gaps within existing services including confusion over who has responsibility for AT, different competing demands on resources in dementia and lack of joint working.

## Discussion

In our study, all of our participants had some knowledge, or experience of AT in a variety of settings. People with dementia and their families gained their knowledge from personal experience rather than from health and social care professionals; sadly most reported feelings of being unsupported for significant periods of time after diagnosis. GPs acquired knowledge via experiential learning rather than formal training. People with dementia, their family carers and professionals commented on the lack of clear information pathways for AT, although both groups highlighted the important role of well-established, voluntary organisations. Despite GPs' acknowledgement of their central role in dementia care, AT was not a routine part of their consultations with people with dementia. The issue of access to AT highlighted deficits in the wider community care system, with all professionals undertaking a ‘little bit of dementia’ in contrast to the management of other long-term care conditions, for example diabetes, where a specialist nurse often acted as a central key worker. Reports^16–18^ have identified the difficulties in current dementia care (lack of local accessible services, lack of integration, underfunding of community services and lack of a single point of access) which make it difficult for people with dementia and their carers to navigate access to services and support. This diffusion of responsibility was also reflected in discussions about the commissioning of AT, with a lack of integration between health and social care systems and an urgent need for more integrated working.

This study includes GPs with a wide range of experiences and responsibilities, a group notoriously difficult to recruit to research. This, combined with the experiences of people with dementia and their family carers, enables an exploration of the issues related to AT in dementia from multiple perspectives. However, our study has several limitations. This study was conducted in one geographical region of England, the results may not be generalisable to other areas, as access to AT may vary between regions with different commissioning groups and local authorities having different funding priorities. The people with dementia and carers who took part in this study may have been those who were more integrated in to local networks and more likely to use, or be open to the use of, AT. It is possible that those who are more socially isolated or not engaged in local support networks may face additional challenges in accessing and using AT. Additionally, this study included only one professional group, GPs; further research is needed to seek the perspectives of the wider health and social care team for example, memory assessment and management services and social services, who also play an important role in supporting families to access AT.

Although our study focused on one aspect of dementia care, AT, many of the issues we identified are illustrative of wider deficiencies in the current community care system in England. Our findings highlight longstanding concerns that both the current and future medical workforce are inadequately prepared to care for our older population. In England, the 2007 National Audit Office report on dementia found that the majority of GPs surveyed lacked the knowledge and confidence to provide care for people with dementia.^19^ Similar deficiencies exist in undergraduate medical education, with a wide range of teaching content delivered^20^ and a lack of a nationally agreed university curriculum for this topic.^21^

The GPs in our study felt that dementia should be considered a long-term ‘chronic illness’; too much emphasis was still placed on a ‘medical model,’ and search for an elusive cure, rather than the delivery of high-quality, person-centred care.^22^ ^23^ Participants suggested a shared care approach with clearly designated professional responsibilities, which is routinely used in other chronic illness care such as diabetes, together with a single point of access for information and support, such as a dementia advisor or nurse specialist, as a means to help people navigate the current chaos. This concurs with findings from recent international research on dementia care^24^ ^25^ and earlier work which explored barriers to telecare integration in chronic disease care.^26^ A case management approach, where a central care/case manager co-ordinates care between professional groups, has been shown to be effective in some chronic conditions.^27^ However, despite promising results from early trials,^28^ ^29^ systematic reviews have found little clinical or cost-effectiveness evidence to support the widespread implementation of case management in dementia^30^ and a recent UK study struggled to implement it within the current care system.^31^

We identified wider governance concerns around the maintenance and future provision of AT. This has recently been highlighted in a study exploring how suppliers could better work with older people; it found a considerable need for ‘personalised’ AT solutions, which required constant refinement to meet individual user needs, over and above the standard devices provided.^32^ Our study shows that awareness and use of AT in dementia care is slowly increasing but that the market appears to be consumer-led despite a sustained policy push in the UK for a health/social care led system.^16^ ^33^ GPs, and all doctors involved in the care of older people, should be equipped with the relevant knowledge to ensure their patients receive appropriate information and support to enable them to live independently for as long as possible. There will always be a tension between the need to collect high-quality evidence about the effectiveness of interventions, which is time consuming, and the need to respond to immediate real world issues. Further research is required that explores more timely access to AT in dementia and information on the types of devices people with dementia and their families find useful.
